# THE ROLE OF SENSE OF PURPOSE IN HEALTH NAVIGATION AMONG MARGINALIZED OLDER ADULTS

**DOI:** 10.1093/geroni/igad104.3663

**Published:** 2023-12-21

**Authors:** Ameesha Narine, Emily Mroz, Shubam Sharma

**Affiliations:** Kennesaw State University, Kennesaw, Georgia, United States; Yale University, New Haven, Connecticut, United States; Kennesaw State University, Kennesaw, Georgia, United States

## Abstract

A sense of purpose is a central, self-organizing life aim that guides choices and behaviors . In older adulthood, having a sense of purpose is linked with positive health. Understanding how older adults who identify as socially marginalized (e.g., racially/ ethnically diverse, LGBTQ+) leverage purpose to navigate their health, for example, when setting health values, making health decision, or adapting to health challenges may support development of tailored resources to combat social disparities embedded in healthcare systems.. This study used qualitative thematic analysis (Braun & Clarke, 2012). to explore the role of purpose in marginalized older adults’ current health navigation and including how purpose and health navigation change across adulthood. Nineteen older men and women who identified as socially marginalized (Mage = 66.5) participated in semi-structured interviews Analyses revealed that older adults draw on purpose to guide health by maintaining personally meaningful, health-related (e.g., health education, caregiving) vocations into late life and by modeling positive health behaviors for others . Further, the role of purpose in health navigation changed across their adulthoods: participants described shifts from adversity-driven purpose to wellness-focused purpose, translating early adversity into current health motivation, and finding space for their authentic purpose when health was considered to stabilize. Findings demonstrate that, for marginalize older adults, their relationship with their own health may improve across the lifespan as they overcome adversity and health becomes enmeshed in purpose. Findings support refining health interventions to account for the role of purpose for older adults who have faced undue social hardship.

